# A reirradiation workflow for managing patients with treatments planned in a multi-TPS environment

**DOI:** 10.1016/j.tipsro.2026.100377

**Published:** 2026-01-07

**Authors:** Jason Vickress, Anders Celinski, Barbara Millman, David A. Palma, Donna H. Murrell

**Affiliations:** a Verspeeten Family Cancer Centre, London Health Sciences Centre, London, ON, Canada; b Department of Oncology, Schulich School of Medicine & Dentistry, Western University, London, ON, Canada

**Keywords:** Reirradiation, Workflow management, Clinical efficiency, Treatment planning, Dose accumulation

## Abstract

Reirradiation is becoming more common. Safe and effective implementation relies on accurate dose accumulation methodology, complex treatment planning, and multidisciplinary communication. Clinical workflow guidance to address these challenges is needed. Herein we report a TPS-agnostic reirradiation workflow, developed at our institution, that facilitates comprehensive cumulative dose evaluation and can improve safety and efficiency in reirradiation programs.

## Highlights

• Reirradiation is increasing. Dedicated workflows, like those described here, are required to manage these complex cases.
• A TPS-vendor agnostic workflow for dose accumulation and evaluation is described.

## Introduction

As clinical demand for reirradiation increases, there is much attention focused on understanding and harmonizing approaches to this treatment [1], [2], [3]. Compared to first course radiotherapy, reirradiation is complex, with higher risk for toxicity due to cumulative doses. Achieving an appropriate radiotherapy plan often requires a highly personalized compromise between competing goals in target coverage vs. organ-at-risk (OAR) sparing. This relies on accurate dose accumulation and multidisciplinary communication to ensure alignment with the treatment intent.

Reirradiation treatment planning and dose accumulation methodology are recognized as key challenges in the field [3], [4]. Requirements to calculate equieffective dose (e.g. equivalent dose in 2 Gy per fraction, EQD2Gy) and map it over time necessitate new software functionality not previously incorporated into treatment planning systems (TPS), which considered only individual courses of radiotherapy [5]. There is also little guidance on how to approach creating a new plan that adequately accounts for previous dose. Together, these challenges result in iterative manual processes for treatment planning that are time-consuming and inefficient.

Safe clinical implementation of reirradiation also faces logistical challenges. There is not yet functionality to identify patients undergoing reirradiation within standard clinical workflows and centres are left to develop their own solutions [5]. Secondly, access to prior radiotherapy treatment records can be difficult, particularly if located in different software systems or at an external centre. Data collation is further complicated when records are in alternative electronic formats (e.g. pre-DICOM era) or in print/film. Finally, robust multidisciplinary communication systems for the special considerations inherent in reirradiation treatment planning and delivery are urgently needed. The development of workflow guidance specific to reirradiation has been identified as an important unmet clinical need [3], [4].

Software capabilities to handle these unique reirradiation hurdles are limited, though early solutions are emerging. Workflows based on clinically-available software by Varian Medical Systems – Radiation Oncology Information System (ROIS) = ARIA, TPS = Eclipse, and the Eclipse Scripting Application Programming Interface (ESAPI) – have been demonstrated [6], [7], [8], [9], as well as modifications using in-house Matlab tools [10] and other approaches using Pinnacle [11], [12]. A workflow within a research version of RayStation treatment planning software has also been described [13]. Notably, these solutions are built on single-vendor TPS platforms, and importing RT data created in other systems can be difficult.

Herein we report a clinically useful universal reirradiation workflow from our institution experienced with high volume and complex reirradiation. We treat approximately 5200 radiation courses per year; one quarter of those are screened via this workflow with a tenth ultimately resulting in a formal dose accumulation. This workflow is TPS vendor agnostic and addresses the key challenges in treatment planning and dose accumulation.

## Workflow

### Identification

**Fig. 1:**
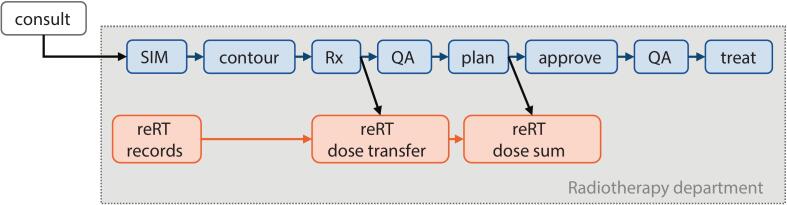
General reirradiation workflow, implemented at our institution via standard Care Path (blue tasks) with reRT specific chain of activities (orange tasks). SIM=CT-Simulation, QA = quality assurance.

Patients who received previous radiotherapy are primarily identified by the Radiation Oncologist on the radiotherapy booking form. A reRT-specific chain of activities is then added to the main Care Path (Fig. 1) when the patient is entered into the ROIS (ARIA, Varian Medical Systems, Inc) for CT-Simulation. This is also added whenever the patient has records of previous RT in the ROIS, or if any team member becomes aware that a patient has received prior radiotherapy. The reRT chain is comprised of 3 tasks: reRT records, reRT dose transfer, and reRT dose sum.

### Record collection

The reRT records task is available before CT-Simulation and initiates collection of relevant prior radiotherapy records. For patients previously treated at outside institutions, record requests are sent and received via direct communication, and secure data transfer (i.e. file transfer protocol [FTP]). After requested files are received and verified, DICOM-RT formatted files are imported into MIM (GE Healthcare), our institution’s Picture Archiving and Communication System (PACS) whereas other files (e.g. PDFs, images of scans, etc.) are imported into the ROIS as documents. For patients treated locally, records need to be unarchived and made available. All DICOM-RT data are sent to a local PACS server, which allows data to be collected and compared from multiple treatment planning systems. A neutral MIM PACS system was required because our institution has patients previously planned in Eclipse, Pinnacle, Precision (Accuray), and RayStation TPS.

### Initial evaluation, dose transfer, and feasibility analysis

The reRT dose transfer task is available after CT-Simulation and contouring are complete, and an initial prescription is entered. At this point, there are two options to cancel the remaining reRT tasks: (1) the physician indicates they do not require cumulative dose evaluation for the clinical context (e.g. following a clinical trial protocol not based on dose accumulation evaluation, low dose prescriptions such that full overlap would not exceed OAR limits, etc.; or (2) the physicist determines there is no risk for high dose overlap between plans (e.g. previous pelvic irradiation and current plan is for brain metastasis). If neither option is applicable, the case proceeds for dose transfer and feasibility analysis.

**Fig. 2:**
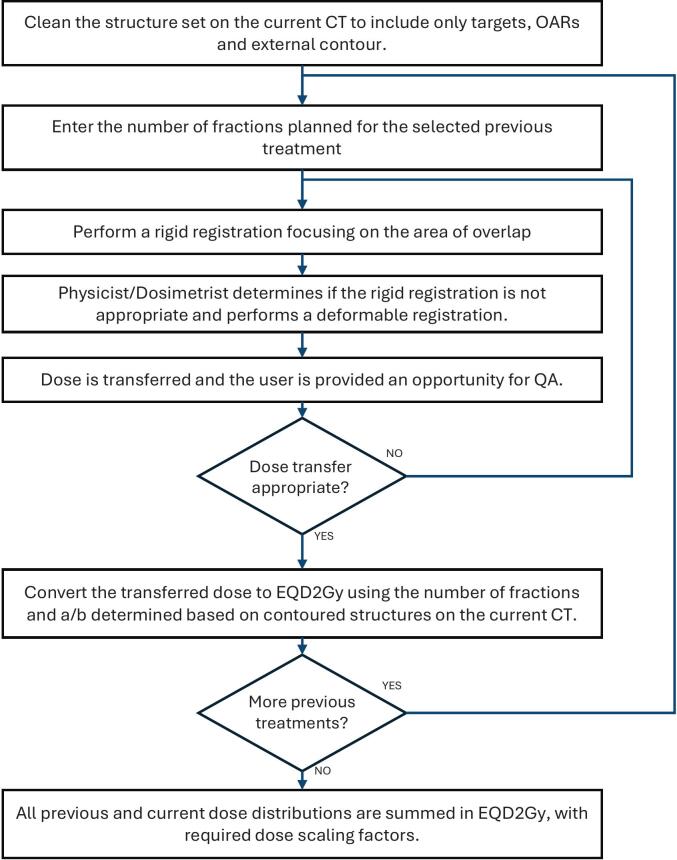
Flow chart describing the semi-automatic workflow in MIM. It requires the CT, structures and dose distribution for all previous courses of treatment, and only the structure set and CT for the current course.

Dose accumulation is primarily performed in MIM using a semi-automated workflow described in Fig. 2. Previous dose is mapped to the current planning CT using rigid or deformable image registration (DIR). If DIR is required, the free-form intensity-based deformable registration from MIM is applied as it is optimized for CT-CT registrations [14]. Registration quality is context-driven and evaluated on a case-by-case basis as discussed by Murr et al [15]. Physical dose is converted to EQD2Gy using the RTst file from the current plan and standard ⍺/β ratios. Notably, the previous plan contours are not used for EQD2 calculation and do not require curation. If relevant, dose scaling factors (DSF) are applied and multiple previous treatments are summed together. If the registration is unsuitable (e.g. surgical intervention resulting in missing organs, incompatible positioning changes such as supine and then prone breast, etc.), or if DICOM data are not available, point dose analysis for relevant OAR is performed using a commissioned Excel spreadsheet and the best available information in the patient record. In these cases, the previous OAR contours are reviewed for accuracy by the Dosimetrist and/or Radiation Oncologist, as needed. A feasibility analysis is performed to determine if the desired planning objectives for target coverage and OAR sparing can be achieved; and if not, what compromises are required to meet accumulated dose objectives. We have established institutional standards for tissue recovery factors, ⍺/β ratios, and cumulative OAR dose constraints (see Supplementary Material), though physicians may request alternate values in their prescription document.

### Treatment planning

Physical dose objectives are created manually or via an automated workflow to support treatment planning, as described below.

**Fig. 3:**
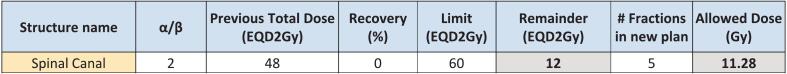
Point dose methodology for determining dose/fractionation constraints in a new plan in order to meet cumulative dose constraints. The shaded columns are calculated; the user inputs data into the other cells.

The point dose method evaluates the total EQD2Gy dose distribution near current targets and uses max point doses (D0.01 cc) to determine what remaining dose is allowable while keeping the cumulative dose within limits. This is a conservative estimate assuming maximum dose overlap, and can be used if there is almost complete overlap or if a point dose constraint is likely to be achieved without compromising the PTV. An example calculation is shown in Fig. 3.

**Fig. 4:**
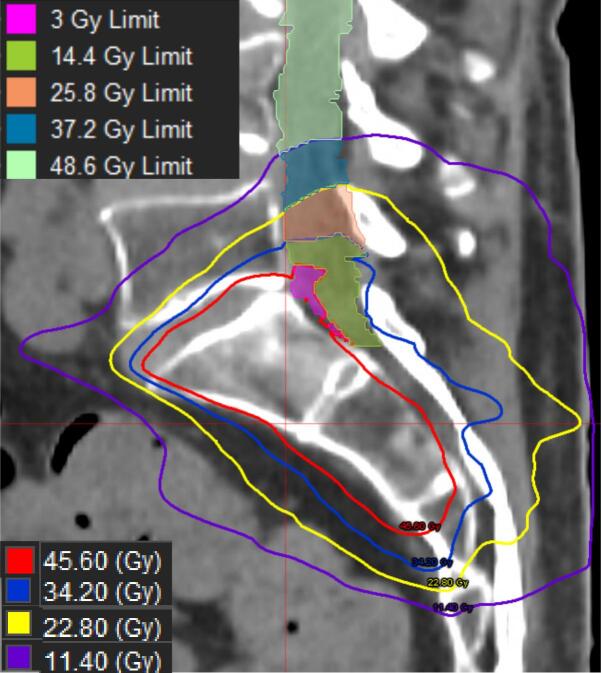
Example of optimization structures with a P = 57 Gy, R = 60 Gy, A = 60 Gy with isodose lines and derived optimization structures for S1-S5 (S1 = pink, S2 = green, S3 = orange, S4 = blue, S5 = pale green). All doses are in EQD2Gy(2) for illustration; the workflow creates limit structures in physical dose for the fractionation scheme prescribed.

Alternatively, optimization structures can be generated based on prior dose distributions to produce dose gradients that limit cumulative OAR doses while maximizing dose to the target. This method is useful when there is high dose overlap and carefully sculpted dose distributions are required to balance tumor coverage and OAR sparing. Manual structure generation can be very time consuming and error prone; to avoid this we created an automated method with a custom MIM workflow described below and illustrated in Fig. 4.

• P, is the Dmax from the summed previous treatments in EQD2Gy to the OAR (within the region near the target), including any DSF applied.
• R is the prescription dose in EQD2Gy for the nearby targets.
• A is the dose constraint for the structure in EQD2Gy.
• N is the number of optimization structures to be created (more structures = smoother gradient; typically N = 3)

If (P + R) < A, no optimization structures are created because full prescription dose can be delivered to the OAR without exceeding the constraint. If (P + R) > A then optimization structures are required to create a dose gradient.

1. Calculate D1 = P − d. The value of d can be calculated as d = ((P + R)-A)/N.
2. Create an isodose line for D1 called S1. For optimization, assume every voxel in the isodose line D1, received a dose of P. The limit for this structure is equal to A – P.
3. For the next structure calculate D2 = D1 – d, and create an isodose line for D2 called S2.
4. Subtract the structure S1 from S2. Now assume every voxel in S2 received the value of D1. The limit for this structure is equal to A – D1.
5. For the next structure calculate D3 = D2 – d, and create an isodose line for D3 called S3.
6. Subtract the structure S1 and S2 from S3. Now assume every voxel in S3 received the value of D2. The limit for this structure is equal to A – D2.
7. The result is three structures S1, S2 and S3, that are used to limit the dose to P, D1 and D2 respectively. An example is illustrated in Fig. 4.
8. Any remaining voxels in the OAR could be planned without considering the previous treatment and use the appropriate dose constraint.

The automated method works for each OAR separately and generates N optimization structures with physical dose limits in the new plan’s fractionation scheme. The optimization structures are provided to the Dosimetrist to help in optimization and generate a treatment plan that would be within desired cumulative dose constraints when added to the previous treatment. This process is conservative with each structure limiting the dose based on the max dose within itself. You can further maximize PTV coverage by increasing the number of optimization structures, at the cost of planning complexity.

In addition to cumulative dose constraints, Dosimetrists have several reirradiation-specific considerations in plan set up. For serial organs, choosing beam geometries that avoid prior high doses to OARs of concern can be sufficient, but it may also be necessary to completely avoid the previously irradiated area. Alternatively, other cases may intentionally overlap doses to maintain sparing of previously spared parallel organs. For all cases, OAR sparing is prioritized over conformity.

### Dose summation

The reRT dose sum task becomes available when the final treatment plan is complete. If MIM was used for dose transfer, then the new plan RTdose is exported to MIM and opened within the dose transfer session. The semi-automated workflow is re-initiated for equieffective dose conversion using the same RTst and ⍺/β ratios, and summed with the previously calculated EQD2Gy doses. A custom report is created including administrative information, cumulative DVH, dose parameters in EQD2Gy, scorecard with cumulative dose objectives, and screenshots of the 3D EQD2Gy isodose distribution. If the Excel spreadsheet was used, it is updated with final point doses achieved in the current plan and the resulting accumulated dose calculation.

### Communication and documentation

A Reirradiation Evaluation and Planning (REP) document is used throughout the workflow for multidisciplinary communication on reRT details (See Supplementary Material). The document is launched at the reRT records task and auto-populates with details of prior radiotherapy delivered via the ROIS. Radiotherapy delivered outside of the ROIS (historical, outside institution, etc.) is entered manually. This information is optionally available to the physician to review as they consider a treatment strategy. Technical information on the dose accumulation approach used is also recorded, including: reRT type and overlap details, plan IDs included, registration type (if applicable), location of original calculation files, and OAR of concern for the new plan. Treatment planning approach, optimization structures/constraints, and discussions between the team on dose objective trade-offs are also recorded. Considerations for treatment delivery are also noted; for example, including an isodose line as a contour to assist with matching and sparing an OAR of concern.

### Training

All staff were thoroughly trained in their respective roles in this workflow. Physicists and Dosimetrists required a comprehensive training program to support the reirradiation treatment planning and dose accumulation work. Some aspects of this included: educational group sessions and one-on-one training in reirradiation principles, image registration, software tools, EQD2 calculation, and dose accumulation. We also developed a curated database of training cases varying in complexity and including multiple body sites, prescriptions, and dose distributions for offline practice. During implementation, we partnered novice with experienced team members for support. We also triaged cases based on complexity to balance training opportunity with specialist demand and efficient patient care.

## Discussion

We have described a TPS-agnostic reirradiation workflow that facilitates comprehensive cumulative dose evaluation and can improve safety and efficiency in reirradiation programs. The default process to include patients into the reRT chain whenever there is prior RT prevents missed prior courses and incorrect assumptions about potential overlap; however, it also increases screening workload. Initiating the record collection task early allows time to retrieve and review records from external sites and/or internal archives. This information then supports dose transfer, feasibility analysis, and achievable strategy development prior to the case transfer to a Dosimetrist. Important benefits of this sequencing are a reduction in futile planning attempts and decreased treatment delays due to planning complexity. Still, initiating the process closer to consult could offer additional gains, such as optimizing CT-Simulation decisions. Feasibility analysis, proactive new plan dose constraint development, and optimization structure creation have minimized the number of iterations to determine an appropriate cumulative dose and improved ease of treatment planning for these complex cases. Finally, the REP document records decision-making for dose accumulation and treatment planning as well as technical parameters in a forum available to all team members. This work contributes to understanding current clinical approaches to reirradiation management and builds toward addressing unmet needs in workflow guidance, treatment planning, and dose accumulation strategies for reirradiation.

## Declaration of competing interest

The authors declare the following financial interests/personal relationships which may be considered as potential competing interests: [David Palma reports a relationship with Ontario Institute for *Cancer* Research that includes: funding grants. If there are other authors, they declare that they have no known competing financial interests or personal relationships that could have appeared to influence the work reported in this paper].
