# Supplementary material for: A reirradiation workflow for managing patients with treatments planned in a multi-TPS environment
Source: Tech Innov Patient Support Radiat Oncol. 2026 Jan 7;37:100377. doi: 10.1016/j.tipsro.2026.100377 (PMC12830171; doi:10.1016/j.tipsro.2026.100377)
Supplement: Supplementary Data 1 [file mmc1.docx]

# Supplementary Material – Institutional Standards

**CUMULATIVE DOSE OBJECTIVES**

*Serial organs*

| **Organ** | **⍺/β** | **Ideal**  **D0.01cc (EQD2Gy)** | **Variation**  **D0.01cc (EQD2Gy)** |
| --- | --- | --- | --- |
| Brain | 2 | 70 |  |
| Brainstem | 2 | 54 | 60 |
| Chiasm | 2 | 54 | 60 |
| Optic Nerve | 2 | 54 | 60 |
| Spinal Cord | 2 | 50 | 54 |
| Cauda | 2 | 60 |  |
| Plexus | 3 | 66 |  |
|  |  |  |  |
| Orbit | 3 | 45 |  |
| Mandible | 3 | 75 |  |
| Esophagus | 3 | 65 | 75 |
| Carotid artery | 3 | 100 | 125 |
| Heart | 3 | 100 |  |
| Great Vessels | 3 | 100 | 144 |
| Trachea  Bronchus | 3 | 80 | 98 |
| Chest wall  Ribs | 3 | 100 | 183 |
|  |  |  |  |
| Stomach | 3 | 60 |  |
| Small Bowel  Duodenum | 3 | 54 | 60 |
| Sigmoid/Colon  Rectum | 3 | 80 | 90 |
| Bladder | 3 | 80 | 92 |
| Femur | 3 | 70 |  |

*Parallel organs*

| **Organ** | **⍺/β** | **Dose Constraint** |
| --- | --- | --- |
| Lung - GTV | 3 | V14.7EQD2Gy < 37%*  Dmean < 14.7 EQD2Gy |
| Liver - GTV or CTV | 3 | Ideal Dmean < 15 EQD2Gy  Variations:  Dmean < 32 EQD2Gy (non cirrhotic) †  Dmean < 30 EQD2Gy (CP class A) †  Dmean < 24 EQD2Gy (CP class B7) † |
| Liver | 3 | 800 cc < 15 EQD2Gy ‡ |
| Bilateral Kidneys | 3 | Dmean < 18 EQD2Gyˆ |
| Single kidney | 3 | If mean dose to 1 kidney >18 EQD2Gy, then V6EQD2Gy (remaining kidney) < 30%ˆ |

* 14.7 EQD2Gy is the equieffective dose to 20 Gy in 30 fractions.

† uninvolved liver MLD from ASTRO Clinical Practice Guideline (standard fx) PRO 2022

‡15 is a physical dose constraint for 3-5 fraction schemes; adjusting to EQD2Gy is 18-24 EQD2Gy.

ˆQUANTEC constraints, Dawson et al IJROBP 2010

**DOSE SCALING FACTORS (DSF)**

| **Time from last day of previous treatment** | **DSF** |
| --- | --- |
| >6 months | 0.9 |
| 1-5 years | 0.8 |
| 5+ years | 0.7 |
