# Supplementary material for: A reirradiation workflow for managing patients with treatments planned in a multi-TPS environment
Source: Tech Innov Patient Support Radiat Oncol. 2026 Jan 7;37:100377. doi: 10.1016/j.tipsro.2026.100377 (PMC12830171; doi:10.1016/j.tipsro.2026.100377)
Supplement: Supplementary Data 2 [file mmc2.pdf]

| Reirradiation Evaluation and Planning (REP) | |
|---|---|
| | NAME: *[This section is auto-populated]* |
| | ID: *[This section is auto-populated]* |
| | RO: *[This section is auto-populated]* |
| | Date: *[This section is auto-populated]* |

**Previous dose delivered via Aria:**

*[This section is auto-populated]*

**Notes about above doses or information about previous dose delivered outside of Aria or at an external site:**

**STOP FORM HERE BECAUSE**

- ☐ **Physics determined no risk of high dose overlap**
- ☐ **RO does not require dose accumulation for clinical situation**

## DOSE ACCUMULATION ASSESSMENT

☐ **3D dose accumulation**

DICOM data exists and registration is acceptable. See MIM for dose accumulation session(s).

| | Reirradiation type | Evaluation Type |
|---|---|---|
| ☐ | Overlap of irradiated volumes | DVH and dose distribution (isodoses)<br>near-max and volume dose objectives |
| ☐ | No overlap<br>Cumulative dose concern | Dose distribution and DVH for specific organ<br>volume dose objectives |
| ☐ | No overlap<br>No cumulative dose concern | Screenshot uploaded<br>no further evaluation |

| Plan ID | Registration (R or D)* | Notes |
|---|---|---|
| | Select | |
| | Select | |
| | Select | |
| | Select | |
| | Select | |
| | Select | |
| | Select | |
| | Select | |

*R=rigid image registration, D=deformable image registration, N/A = not applicable and not included in dose accumulation

| **MIM SESSION for dose transfer** | |
|---|---|

☐ **Point dose accumulation**

DICOM data unavailable or acceptable registration cannot be achieved - dose accumulation performed in Excel sheet. See ARIA document(s) “Previous Trt BED Calc” for details and analysis.

**Organs of concern for new plan**

| Structure | Dose Scaling Factor | New # fractions | **Allowed dose (Gy)** |
|---|---|---|---|
| | | | |
| | | | |
| | | | |
| | | | |
| | | | |
| | | | |

| Additional planning notes: |
|---|
| |

**Considerations for treatment delivery**

- ☐ no special considerations for matching
- ☐ journal note IGRT instructions to unit MRTs
- ☐ send isodose line as contour to assist with match
- ☐ require physics/RO presence for image match (must add task)

**Additional communication notes from any discipline:**
